# Rates and predictors of hypoglycaemia in 27 585 people from 24 countries with insulin‐treated type 1 and type 2 diabetes: the global HAT study

**DOI:** 10.1111/dom.12689

**Published:** 2016-06-20

**Authors:** K. Khunti, S. Alsifri, R. Aronson, M. Cigrovski Berković, C. Enters‐Weijnen, T. Forsén, G. Galstyan, P. Geelhoed‐Duijvestijn, M. Goldfracht, H. Gydesen, R. Kapur, N. Lalic, B. Ludvik, E. Moberg, U. Pedersen‐Bjergaard, A. Ramachandran, on behalf of the HAT Investigator Group

**Affiliations:** ^1^ Diabetes Research CentreUniversity of LeicesterLeicesterUK; ^2^ Al Hada Military HospitalTaifSaudi Arabia; ^3^ LMC Diabetes and EndocrinologyTorontoCanada; ^4^ University Hospital ‘Sestre Milosrdnice’ZagrebCroatia; ^5^ Julius Clinical/Julius CenterUMC UtrechtZeistthe Netherlands; ^6^ Department of General Practice and Primary Health CareUniversity of HelsinkiHelsinkiFinland; ^7^ Endocrinology Research CenterMoscowRussian Federation; ^8^ Medical Center HaaglandenThe Haguethe Netherlands; ^9^ Clalit Health ServicesTel AvivIsrael; ^10^ The TechnionHaifaIsrael; ^11^ Novo Nordisk A/SSøborgDenmark; ^12^ Faculty of Medicine, Clinic for Endocrinology, Diabetes and Metabolic Diseases, Clinical Center of SerbiaUniversity of BelgradeBelgradeSerbia; ^13^ Rudolfstiftung Hospital and Medical University of ViennaViennaAustria; ^14^ Karolinska InstitutetStockholmSweden; ^15^ Nordsjællands Hospital HillerødHillerødDenmark; ^16^ India Diabetes Research Foundation and Dr A Ramachandran's Diabetes HospitalsChennaiIndia

**Keywords:** diabetes, global, HAT study, hypoglycaemia, insulin, observational

## Abstract

**Aims:**

To determine the global extent of hypoglycaemia experienced by patients with diabetes using insulin, as there is a lack of data on the prevalence of hypoglycaemia in developed and developing countries.

**Methods:**

This non‐interventional, multicentre, 6‐month retrospective and 4‐week prospective study using self‐assessment questionnaire and patient diaries included 27 585 patients, aged ≥18 years, with type 1 diabetes (T1D; n = 8022) or type 2 diabetes (T2D; n = 19 563) treated with insulin for >12 months, at 2004 sites in 24 countries worldwide. The primary endpoint was the proportion of patients experiencing at least one hypoglycaemic event during the observational period.

**Results:**

During the prospective period, 83.0% of patients with T1D and 46.5% of patients with T2D reported hypoglycaemia. Rates of any, nocturnal and severe hypoglycaemia were 73.3 [95% confidence interval (CI) 72.6–74.0], 11.3 (95% CI 11.0–11.6) and 4.9 (95% CI 4.7–5.1) events/patient‐year for T1D and 19.3 (95% CI 19.1–19.6), 3.7 (95% CI 3.6–3.8) and 2.5 events/patient‐year (95% CI 2.4–2.5) for T2D, respectively. The highest rates of any hypoglycaemia were observed in Latin America for T1D and Russia for T2D. Glycated haemoglobin level was not a significant predictor of hypoglycaemia.

**Conclusions:**

We report hypoglycaemia rates in a global population, including those in countries without previous data. Overall hypoglycaemia rates were high, with large variations between geographical regions. Further investigation into these differences may help to optimize therapy and reduce the risk of hypoglycaemia.

## Introduction

Insulin therapy is essential for the treatment of type 1 diabetes (T1D), and is often required for people with type 2 diabetes (T2D). Hypoglycaemia remains a limiting factor in achieving good glycaemic control 1 and recent diabetes treatment guidelines highlight the need for personalized glycated haemoglobin (HbA1c) targets to balance reductions in hyperglycaemia with the potential risks of hypoglycaemia 2, 3.

Previous studies in hypoglycaemia have been focused on the safety and efficacy of particular drugs 4, 5, 6, 7. Data regarding hypoglycaemia rates obtained from randomized controlled trials, as opposed to observational studies, must be interpreted with caution as these often exclude older patients, and those with recurrent hypoglycaemia, very poor glycaemic control (HbA1c >10%), or concomitant medical conditions, even though these variables are often seen in the clinic. In addition, such studies are conducted under controlled conditions, with regular contact and follow‐up between patients and trial physicians, and are often of a treat‐to‐target design to meet regulatory requirements 8. Both this selection of patients and trial design are likely to influence the observed rate of hypoglycaemia.

Observational studies and surveys conducted thus far have reported somewhat higher non‐severe hypoglycaemia frequency ranges of 3.5–7.2 events/month for T1D 1, 9, 10, 11 and 0.8–4.0 events/month for T2D 1, 9, 10, 11, 12, 13; however, these studies were primarily retrospective or cross‐sectional studies (leading to potential recall bias), conducted online (restricting participation to those who have access to and ability to use the internet, which is a potential source of selection bias, particularly for older patients), and have thus far been limited to North America and Europe.

Beyond hypoglycaemia rates, it is also important to examine factors associated with hypoglycaemia to identify higher‐risk patients and to tailor treatment appropriately, particularly with regard to setting realistic targets for glycaemic control. Large‐scale studies of hypoglycaemia rates in clinical practice are therefore required to determine any factors associated with hypoglycaemia, and to ascertain the real‐life magnitude and impact of hypoglycaemia rates, particularly outside Europe and North America.

The aim of the present study, the HAT study, was to examine the impact of hypoglycaemia in an insulin‐using global patient population in an epidemiological observational study covering a 6‐month retrospective and a 4‐week prospective time period.

## Research Design and Methods

### Study Design

This study was a non‐interventional, multicentre, 6‐month retrospective and 4‐week prospective study of hypoglycaemic events across 2004 sites in 24 countries in six regions (Eastern Europe: Bulgaria, Croatia, Czech Republic, Hungary, Poland, Romania, Russian Federation, Serbia, Slovakia and Slovenia; Latin America: Argentina and Mexico; Middle East: Israel, Lebanon and Saudi Arabia; Northern Europe/Canada: Austria, Canada, Denmark, Finland, Germany, the Netherlands and Sweden; Russia: Russian Federation; Southeast (SE) Asia: India and Malaysia) using self‐assessment questionnaires (SAQs) and patient diaries (for 28 days). The site selection was a convenience sample. The study was rolled out over a period of 1 year from 2012 to 2013 in a staggered fashion (start times varied by country). The study protocol and assessments were conducted in accordance with the Declaration of Helsinki (2004) and the International Conference on Harmonisation Guidelines for Good Clinical Practice (1996), and approved by country‐specific regulatory agencies. All study materials were translated into local languages, and data obtained were translated back into English for analysis.

### Study Population

Consecutive patients were enrolled during a routinely scheduled clinical consultation with their healthcare provider. Eligible patients were aged ≥18 years at baseline, with T1D or T2D treated with insulin for >12 months, who had given informed consent to participate in the study. Exclusion criteria included non‐ambulatory status and illiteracy or other issues resulting in an inability to complete a written questionnaire. Patients were not paid for their participation in the study.

### Assessments

The study comprised a two‐part SAQ. Part 1 was a cross‐sectional assessment used to record baseline demographic and treatment information, as well as the history of severe hypoglycaemia over the last 6 months and non‐severe hypoglycaemia over the previous 4 weeks in the lead up to baseline study entry. Part 2, completed 4 weeks later, evaluated the occurrence of both severe and non‐severe hypoglycaemia over the 4 weeks following baseline study entry. To assist recall, patients were provided with a diary, which was also used to record hypoglycaemic events anonymously. If a patient recorded more hypoglycaemic events using the patient diary than the Part 2 SAQ, the patient diary value was used to calculate prevalence of hypoglycaemia in the 4 weeks after baseline, to compensate for potential underestimates attributable to recall bias.

### Study Objectives

The primary endpoint of the study was the percentage of patients experiencing at least one hypoglycaemic event during the 4‐week follow‐up period. Secondary endpoints included: hypoglycaemia rates, HbA1c level at baseline, relationship between HbA1c and hypoglycaemia, including proportion of patients with HbA1c <7.0% (53 mmol/l) and >9.0% (75 mmol/l) with or without hypoglycaemia, and relationship between hypoglycaemia and factors such as age, fear of hypoglycaemia, disease duration and duration of insulin use. Although the study included both retrospective and prospective collection periods, this report has focused on data obtained in the prospective period, since it may be less prone to recall bias.

### Hypoglycaemia Classification

Categories of hypoglycaemia recorded in the questionnaire and patient diary included non‐severe hypoglycaemia (defined as an event managed by the patient alone), severe hypoglycaemia (defined, based on the American Diabetes Association definition, as any hypoglycaemic event requiring assistance of another person to administer carbohydrate, glucagon or other resuscitative actions 14) and nocturnal hypoglycaemia (any event occurring between midnight and 06:00 hours). A combined measure of any hypoglycaemia, based on the sum of all individual hypoglycaemic events of any categories, was calculated based on diary and questionnaire entries.

### Sample Size

Target sample size was based on the desired level of precision for estimating the percentage of patients experiencing at least one hypoglycaemic event during the observation period. Calculations of the percentage of patients experiencing a hypoglycaemic event and the 95% confidence interval (CI) for various sample sizes indicated that the optimum CI precision would be achieved with a sample size of 12 000. Assuming a SAQ Part 2 responder rate of 37%, the total number of patients to be screened was determined to be ∼32 000.

### Statistical Analyses

All statistical tests were two‐sided and regarded as exploratory, with the criterion for statistical significance set at p < 0.05. No adjustments were made for multiple comparisons.

For the primary endpoint, the percentage of patients experiencing any hypoglycaemia during the observation period was calculated together with the 95% CI for this percentage. For the secondary endpoints of severe or nocturnal hypoglycaemic events, the number and proportion of patients having an event, number of events, follow‐up time (patient‐years), estimated hypoglycaemia rate with corresponding 95% CI, and number of patients missing, was presented for the 4 weeks after baseline.

Univariate negative binomial regression models, based on the completer analysis set (patients who completed the Part 2 SAQ), stratified by country, specifying a log‐transformed exposure time offset term and adjusted for all variables in the model, were used to examine the relationship between hypoglycaemia and the following factors: age in years, gender, HbA1c in mmol/mol and percentage, duration of diabetes in years, duration of insulin therapy in years, type of insulin therapy, frequency of blood glucose testing in average number of checks per day, knowledge of hypoglycaemia (i.e. knowing what hypoglycaemia is before reading the definition in the SAQ introduction), fear of hypoglycaemia, study period (prospective/retrospective) and diabetes type. Given that the majority of analyses were descriptive in nature, no imputation of missing data was performed.

## Results

### Patient Characteristics

A total of 27 585 patients (T1D, n = 8022; T2D, n = 19 563) completed Part 1. Of these, 25 505 patients (T1D, n = 7070; T2D, n = 18 435) completed Part 2 and 23 627 patients (T1D, n = 6822; T2D, n = 16 805) completed the patient diary. Descriptive baseline characteristics of the global population are shown in Table 1. A total of 51.2% of the study cohort were male. Those with T1D were younger than those with T2D (age 42.1 years vs. 60.8 years, respectively), had a longer duration of diabetes (17.6 years vs. 13.7 years, respectively) and, as insulin use in patients with T1D starts at diagnosis, had therefore been using insulin for a longer period than patients with T2D (17.0 years vs. 6.4 years, respectively). Levels of glycaemic control, in terms of HbA1c_,_ were similar in patients with T1D and T2D [7.9% (63 mmol/l) vs. 8.0% (64 mmol/l), respectively]. In total, 44.6% of all patients defined hypoglycaemia by using both symptoms and blood glucose measurements rather than by symptoms alone [T1D, 49.1% (n = 3758); T2D, 42.3% (n = 6231)].

**Table 1:** Characteristics of population.

Characteristic	T1D (n = 8022)	T2D (n = 19 563)
Sex: male/female, %	48/52	53/47
Mean age, years (s.d.; IQR)	42.1 (15.1; 28.0–50.0)	60.8 (10.9; 55.0–69.0)
Duration of diabetes, years (s.d.; IQR)	17.6 (12.0; 9.0–24.0)	13.7 (8.2; 8.0–20.0)
Duration of insulin use, years (s.d.; IQR)	17.0 (12.1; 8.0–24.0)	6.4 (5.6; 3.0–10.0)
HbA1c, mmol/mol (s.d.; IQR)	62.8 (16.2; 55.2–74.4)	64.2 (16.3; 51.9–73.8)
HbA1c*, % (s.d.; IQR)	7.9 (1.5; 7.2–9.0)	8.0 (1.5; 6.9–8.9)
Checks blood sugar levels, n (%)		
Yes	7888 (98.6)	17,858 (91.6)
No	110 (1.4)	1635 (8.4)
Has experienced hypoglycaemia, n (%)		
Yes	7759 (97.4)	15,167 (78.3)
No	159 (2.0)	3272 (16.9)
Not sure	49 (0.6)	940 (4.9)

IQR, interquartile range; s.d., standard deviation.

* Calculated, not measured.

Regional completion rates of the questionnaires and diaries are shown in Table S1 (Supporting Information), with regional baseline characteristics shown in Tables S2 and S3, Supporting Information, for T1D and T2D, respectively. HbA1c levels were numerically higher in regions outside Europe and Canada (Latin America, Middle East, Russia and SE Asia groups), whereas the duration of diabetes and of insulin use were numerically higher in Northern Europe/Canada.

### Reporting of Hypoglycaemia

In the 4 weeks after baseline, 5886 patients with T1D (83.0%; 95% CI 82.1–83.9) and 8580 patients with T2D (46.5%; 95% CI 45.8–47.2) reported experiencing at least one hypoglycaemic event. Estimated annual rates of any hypoglycaemia in the prospective period were 73.3 events/patient‐year (95% CI 72.6–74.0) and 19.3 events/patient‐year (95% CI 19.1–19.6) for patients with T1D and T2D, respectively.

Nocturnal hypoglycaemia was reported in 2768 patients with T1D (40.6%; 95% CI 39.4–41.7), with an estimated rate of 11.3 events/patient‐year (95% CI 11.0–11.6). A total of 2800 patients with T2D (15.9%; 95% CI 15.4–16.5) reported nocturnal hypoglycaemia, with a rate of 3.7 events/patient‐year (95% CI 3.6–3.8).

Overall, 1024 patients with T1D (14.4%; 95% CI 13.6–15.3) and 1635 patients with T2D (8.9%; 95% CI 8.5–9.3) reported a severe hypoglycaemic event. Annual rates of severe hypoglycaemia based on the prospective period were 4.9 events/patient‐year (95% CI 4.7–5.1) and 2.5 events/patient‐year (95% CI 2.4–2.5) for T1D and T2D, respectively. The rates of hypoglycaemia requiring hospitalization during the prospective period were 0.237 events/patient‐year (95% CI 0.198–0.283) for patients with T1D and 0.221 (95% CI 0.196–0.247) for patients with T2D. Rates of hypoglycaemia by age group in the 4 weeks after baseline are shown in Table S5, Supporting Information.

### Regional Differences in Hypoglycaemia Rates

The proportions of patients with T1D experiencing hypoglycaemia were 86.7% (1558/1797) for Northern Europe and Canada, 85.0% (2583/3052) for Eastern Europe, 87.4% (373/427) for Latin America, 72.0% (718/997) for the Middle East, 87.4% (533/611) for Russia, and 54.0% (121/224) for SE Asia. The proportions of patients with T2D experiencing hypoglycaemia were 43.6% (1460/3352) for Northern Europe and Canada, 53.8% (3312/6218) for Eastern Europe, 43.8% (644/1469) for Latin America, 39.1% (1149/2942) for the Middle East, 62.6% (454/726) for Russia, and 41.0% (1561/3811) for SE Asia.

**Figure 1:**
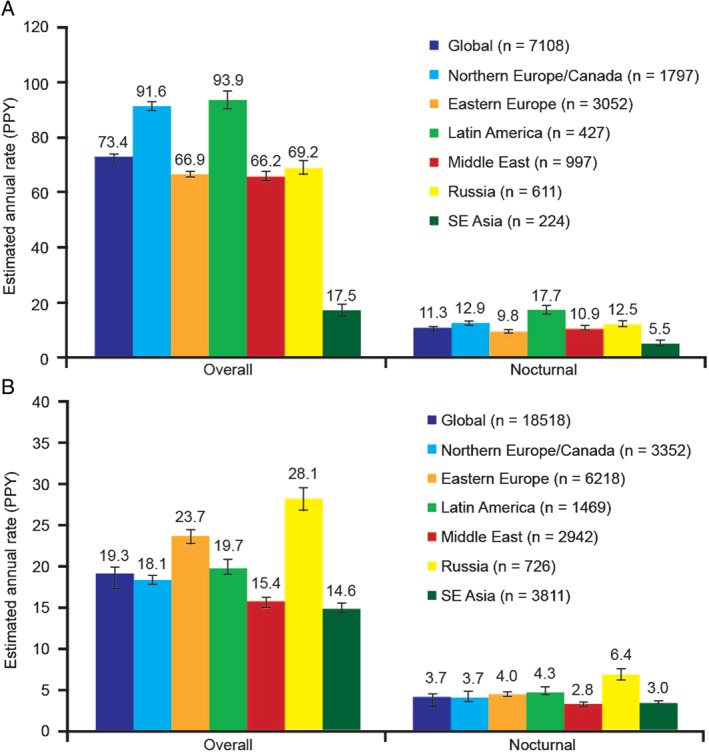
Overall and nocturnal hypoglycaemia rates during the prospective period by geographic region. (A) Patients with type 1 diabetes. (B) Patients with type 2 diabetes. PPY, per patient‐year; SE, Southeast.

Overall and nocturnal hypoglycaemia rates are shown in Figure 1, broken down by region and type of diabetes. Overall hypoglycaemia rates for patients with T1D were highest in Northern Europe and Canada, and Latin America (91.6 and 93.9 events/patient‐year, respectively) compared with rates of ∼70 events/patient‐year for all other regions surveyed, with the exception of SE Asia (17.5 events/patient‐year). Latin America also had the highest rates of nocturnal hypoglycaemia in patients with T1D (17.7 events/patient‐year). Overall hypoglycaemia rates in patients with T2D were consistent across most participating regions, with the highest rates being in Eastern Europe and Russia (23.7 and 28.1 events/patient‐year, respectively). Russia also had the highest rates of nocturnal hypoglycaemia in patients with T2D (6.4 events/patient‐year).

Severe hypoglycaemia rates by region are shown in Table S4, Supporting Information. The highest rates for severe hypoglycaemia in T1D were reported in Latin America and the Middle East, whereas the highest rates for severe hypoglycaemia in T2D were reported in Latin America and SE Asia.

### Factors Associated with Hypoglycaemia

**Figure 2:**
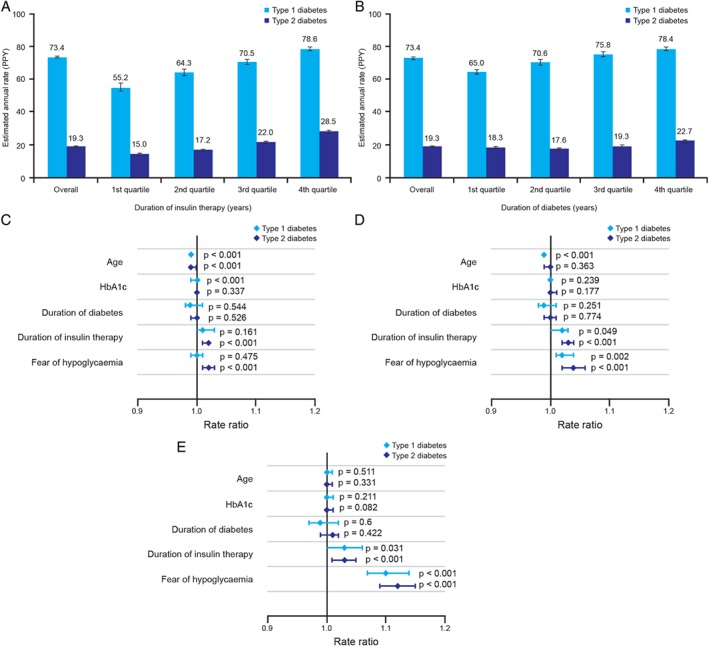
Relationship between estimated rates of any hypoglycaemia and (A) duration of insulin treatment and (B) duration of diabetes. Fully adjusted negative binomial modelling of the associations between patient characteristics and incidence rate ratios for (C) any, (D) nocturnal or (E) severe hypoglycaemia in the global trial population. Part A shows duration of insulin therapy for type 1 diabetes (T1D), lower quartile 7.0 years, median 15.0 years, upper quartile 24.0 years; for type 2 diabetes (T2D), lower quartile 2.0 years, median 5.0 years, upper quartile 9.0 years. Part B shows duration of diabetes for T1D, lower quartile 8.0 years, median 15.0 years, upper quartile 24.0 years; for T2D, lower quartile 8.0 years, median 12.0 years, upper quartile 18.0 years. HbA1c, glycated haemoglobin; PPY, per patient‐year.

A numerical increase in hypoglycaemia rate was observed with increased duration of diabetes and increased duration of insulin therapy for both T1D and T2D (Figure 2).

Fully adjusted negative binomial modelling results for association between any, nocturnal or severe hypoglycaemia and age, HbA1c, duration of diabetes, duration of insulin use, and fear of hypoglycaemia (as indicated on a 10‐point scale), are shown in Figure 2C–E.

Older age was associated with a reduced risk of any hypoglycaemia in patients with T1D or T2D: rate ratio 0.99 (95% CI 0.99; 0.99; p < 0.001). Duration of insulin therapy was associated with overall, nocturnal and severe hypoglycaemia in patients with T2D. Fear of hypoglycaemia was associated both with any hypoglycaemia and nocturnal hypoglycaemia in T2D, whereas severe hypoglycaemia was associated with greater fear of hypoglycaemia in both T1D and T2D.

**Figure 3:**
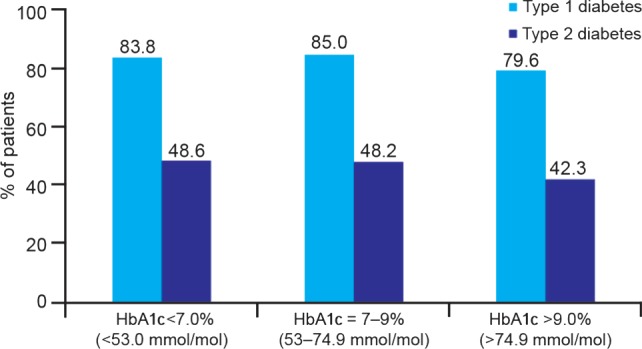
Percentage of patients reporting hypoglycaemia during the prospective period, stratified by glycated haemoglobin (HbA1c) level for patients with type 1 and type 2 diabetes.

To elucidate further the association between HbA1c and hypoglycaemia, the proportion of patients with any hypoglycaemic event by HbA1c level is plotted for T1D and T2D in Figure 3. HbA1c was not found to be a significant predictor of hypoglycaemia.

## Discussion

The present study examined hypoglycaemia prevalence and rates in a large global cohort of insulin‐treated patients with diabetes, including many countries and several regions with no previously published data. Patient‐reported hypoglycaemia in a global population occurred at a higher frequency than previously reported, with marked variations across geographic regions. Patients with T1D in Northern Europe/Canada and Latin America reported the highest rates of any hypoglycaemia. Regional differences in both overall and nocturnal hypoglycaemia were also observed in patients with T2D, with the highest rates being reported in Russia and Eastern Europe and the lowest rates in SE Asia and the Middle East. Differences were also apparent in severe hypoglycaemia rates. Notably, Latin America had the highest rates of severe hypoglycaemia for T1D and T2D. Hypoglycaemia rates were only weakly associated with HbA1c level for T1D and did not appear to be significantly associated with HbA1c level in T2D. There was an association between increased rates of hypoglycaemia and duration of insulin therapy in T2D. No other strong associations between hypoglycaemia and the specified variables were evident in this study.

Limitations of the HAT study include its observational nature and short prospective duration; however, these characteristics allowed a large patient pool from which meaningful observations regarding the real‐life rate and impact of hypoglycaemia could be made. Whilst the HAT study represents an advance on previous studies in estimating the global prevalence of hypoglycaemia, willingness to participate and local literacy rates are likely to have affected the participant characteristics. The simplicity of the questionnaires, although limiting the amount of additional information available for subsequent sub‐analyses, may also have contributed to the high completion rate. The questionnaire did not record insulin regimen or sulphonylurea use, both of which could contribute to regional differences in the rate of hypoglycaemia. Patient diaries were used in the prospective period in addition to the Part 2 SAQ to reduce recall bias. Use of patient‐reported data from the diaries in addition to the SAQ Part 2 may have increased the reliability of data on prevalence of hypoglycaemia but has the potential to overestimate hypoglycaemia rates.

In the present study, as with previous self‐reporting studies, patients were permitted to record a hypoglycaemic episode by either symptoms or blood glucose testing alone, or in combination. This approach represents both a strength and a limitation of the study; it aided the capture of events in which patients forgot or neglected to test blood glucose, did not know the blood glucose concentration threshold for hypoglycaemia, or were unable to test because of a lack of testing devices/materials, but also introduced the potential for confounding because of the subjective nature of the assessment. The lack of newly diagnosed/treated patients (<12 months insulin use) in HAT could affect the observed rates of hypoglycaemia; however, this group is only a small proportion of the total population with T2DM and is unlikely to have a significant effect on the mean values. In addition, the hypoglycaemia event rates are typically lower in this population 15.

We report higher estimated annual rates of hypoglycaemia for both T1D and T2D than previously observed in other studies (mainly randomized controlled trials) 16, 17, 18, 19. In the Veterans Affairs Diabetes Trial, a randomized trial of intensive glucose control in T2D which followed 1791 selected patients for a median of 5.6 years, rates of any symptomatic hypoglycaemic episode were 383–1333 per 100 patient years, whereas rates of severe hypoglycaemia were 3–9 per 100 patient years 16. Results from the present observational study, which included both patients with T1D and T2D who had been using insulin for at least 1 year, and collected data both retrospectively and prospectively, differ by at least one order of magnitude from these data. Such marked differences are likely to be attributable both to the controlled nature of randomized trials and the exclusion of patients who experience recurrent severe hypoglycaemia or hypoglycaemia unawareness (occasionally/never have symptoms associated with low blood sugar measurement), are older, or have other concomitant diseases, from participating in clinical trials 20. Hypoglycaemia rates reported from observational studies are somewhat higher, with rates of 42.9 events/patient‐year and 1.15 severe events/patient‐year for patients with T1D, compared with 16.37 events/patient‐year and 0.35 severe events/patient‐year for patients with T2D in a recent population‐based study 20, whereas the monthly rates of hypoglycaemia were somewhat higher in the DIALOG study, at 6.3/month (T1D) and 1.6/month (T2D) 21. The UK Hypoglycaemia Study, which stratified subjects according to duration of insulin use, reported mild hypoglycaemia rates of 10.2 events/patient‐year and severe hypoglycaemia rates of 0.7 events/patient‐year in patients with T2D using insulin for >5 years 22. A recent meta‐analysis of 46 population‐based studies of people with T2D found a higher prevalence of mild/moderate hypoglycaemia and severe hypoglycaemia in patients using insulin (23 events/patient‐year and one event/patient‐year, respectively) 23. These rates are mostly lower than those seen in the overall HAT study, but are broadly similar to the results we report in the present Northern Europe/Canada cohort; however, there are no other published data as a comparator for most of the regions included in the HAT study. The duration of diabetes reported in the present study was longer than in the 9–12‐month observational study carried out by the UK Hypoglycaemia Study group 22. Considering the duration of disease, HbA1c appeared to be relatively well controlled across the large HAT study cohort. The lack of correlation between HbA1c and hypoglycaemia for T2D in this study may appear to be counter‐intuitive; however, it is important to keep in mind that the nature of the study was non‐interventional and hence the findings might be a true representation of what happens in clinical practice.

The high rates of hypoglycaemia in patients with T1D in Northern Europe/Canada may partly be related to the duration of diabetes and duration of insulin use in this population, which, possibly as a result of better prognosis, were considerably higher than those for other regions (Table S2, Supporting Information), but the high rate of hypoglycaemia in T1D seen in Latin America compared with a region such as Eastern Europe, which has similar baseline population characteristics, may reflect regional differences in diabetes management. Relatively low rates of hypoglycaemia among patients with T1D observed in SE Asia could reflect the relatively low average duration of diabetes (11.6 years) and high HbA1c 8.9% (74 mmol/mol), but could also have a variety of other causes, such as differences in self‐monitoring, and should be interpreted with caution because of the relatively low number of patients (n = 224). While the level of patient access to self‐monitoring of blood glucose equipment was not captured in the present study, 53% of patients with T1D and 38% of those with T2D in SE Asia increased blood glucose monitoring after a hypoglycaemic event, suggesting that access to self‐monitoring was fairly widespread; however, we cannot confirm the proportion of patients with access to self‐monitoring represented in these figures 24. When examining how regional differences in hypoglycaemia in patients with T2D may relate to patient characteristics, it appears that patients in Russia and Eastern Europe were older, and had been receiving insulin therapy for longer than patients in the SE Asia and Middle East cohorts (longer disease duration and longer duration of insulin therapy were associated with increased rates of hypoglycaemia in the HAT study). Nevertheless, these regional differences, particularly when occurring between groups with similar baseline characteristics, may imply lack of knowledge or awareness of hypoglycaemia and indicate that there are opportunities to optimize diabetes care further in those countries with higher hypoglycaemia rates. The weak association between HbA1c and hypoglycaemia for T1D observed in the HAT study is in agreement with a recent trend analysis conducted in a large (n > 35 000) patient cohort, which concluded that the association of HbA1c with hypoglycaemia in T1D has decreased in recent years 25, possibly because patients today are titrated to the limit of hypoglycaemia. These findings suggest that the link between HbA1c and hypoglycaemia may be more subtle than previously anticipated, thereby supporting the call for tailored, individualized treatment based on early response to treatment adjustments 2. The association of hypoglycaemia with duration of insulin therapy in patients with T2D may reflect the progressive nature of this disease, which is accompanied by a need for more intensive insulin therapy and impaired hormonal counter‐regulation 26, 27. The association of fear of hypoglycaemia with any and nocturnal hypoglycaemia in patients with T2D probably arises because patients who experience hypoglycaemia more frequently report greater fear of hypoglycaemia as a result. This association may be particularly true of severe hypoglycaemia, which was associated with greater fear of hypoglycaemia in both patients with T1D and those with T2D. It is envisaged that these observations, together with those previously reported, will aid clinicians in better tailoring insulin treatment for patients with diabetes, particularly in regions where such data were previously unavailable.

Further studies and analyses are required to investigate the reasons for the differences in hypoglycaemia rates between the global regions. The lack of any strong association between hypoglycaemia and conventional predictive factors, generally and across regions, in the present study requires exploration of other domains such as ethnic, cultural and healthcare organizational aspects so as to reduce this very complex and important clinical problem.

## Conflict of Interest

K.K. has acted as a consultant, advisory board member, and speaker for and has received research grants from Novo Nordisk, Eli Lilly, Merck Sharp & Dohme, Bristol‐Myers Squibb, AstraZeneca, Sanofi, Boehringer Ingelheim and Roche. S.A. has no conflicts of interest to disclose. R.A. has provided research support, acted as a consultant and advisory board member, and has received research grants from Novo Nordisk, Janssen, Sanofi, Medtronic, Bristol‐Myers Squibb, AstraZeneca, Takeda, Becton Dickinson, Merck Sharp & Dohme, Boehringer Ingelheim, Regeneron, Eli Lilly, Abbott, Quintiles, ICON, Medpace and GlaxoSmithKline. M.C.B. has acted as a board member for Novo Nordisk and Novartis, and speaker for Novo Nordisk, Eli Lilly, Merck Sharp & Dohme, AstraZeneca, Sanofi, Boehringer Ingelheim and Novartis. C.E.‐W. has received unrestricted educational grants from Novo Nordisk and Pfizer. T.F. has no conflicts of interest to disclose. G.G. has acted on advisory panels and as a board member and speaker for Novo Nordisk, Eli Lilly, Merck Sharp & Dohme, AstraZeneca, Sanofi, Novartis and Takeda. P.G.‐D. has acted as a speaker for and received research grants from Novo Nordisk. M.G. has no relevant conflicts of interest to disclose. H.G. and R.K. are employees of Novo Nordisk. N.L. has no conflicts of interest to disclose. B.L. has acted as an advisory board member and speaker for Amgen, Allergan, AstraZeneca, Bristol‐Myers Squibb, Boehringer Ingelheim, Eli Lilly, GlaxoSmithKline, Merck Serono KGaA, Merck Sharp & Dohme, Novartis, Novo Nordisk, Pfizer, Sanofi, and Servier. E.M. has acted as a speaker for Novo Nordisk, Eli Lilly, Merck Sharp & Dohme, Sanofi, and Boehringer Ingelheim. U.P.‐B. has acted as a consultant, advisory board member, and speaker for and has received research grants from Novo Nordisk, Eli Lilly, Merck Sharp & Dohme, Bristol‐Myers Squibb, AstraZeneca, Sanofi, Boehringer Ingelheim and Roche. A.R. has acted as an advisory board member and speaker for AstraZeneca, Bristol‐Myers Squibb, Novo Nordisk and Sanofi, and has received research grants from Novo Nordisk.

K.K., S.A., T.F., P.G.‐D., H.G., R.K., B.L. and E.M. contributed to the study design. K.K., S.A., R.A., M.C.B., T.F., P.G.‐D., M.G., N.L., E.M. and U.P.‐B. conducted the study and data collection. K.K., R.A., M.C.B., G.G., P.G.D., M.G., H.G., R.K., B.L., U.P.‐B. and A.R. analysed/interpreted the data. K.K., S.A., R.A., M.C.B., C.E.‐W., T.F., P.G.D., M.G., R.K., B.L., E.M. and U.P.‐B. contributed to the writing of the manuscript, which was reviewed by all authors.

## Supporting information


**File S1.** Patient disposition by country.

**File S2.** Baseline characteristics by region of patients with type 1 diabetes.

**Table S1.** Patient disposition by country.
**Table S2.** Baseline characteristics by region of patients with type 1 diabetes.
**Table S3.** Baseline characteristics by region of patients with type 2 diabetes.
**Table S4.** Severe hypoglycaemia rates during the prospective period by geographic region.
