# Supplementary material for: Rates and predictors of hypoglycaemia in 27 585 people from 24 countries with insulin‐treated type 1 and type 2 diabetes: the global HAT study
Source: Diabetes Obes Metab. 2016 Jun 20;18(9):907–15. doi: 10.1111/dom.12689 (PMC5031206; doi:10.1111/dom.12689)
Supplement: Supplementary file 1 — File S1. Patient disposition by country. [file DOM-18-907-s001.docx]

**Distribution of Hypoglycaemic Events for Patients Experiencing Any Hypoglycaemia in the 4 Weeks Before Baseline (Full Analysis Set)**


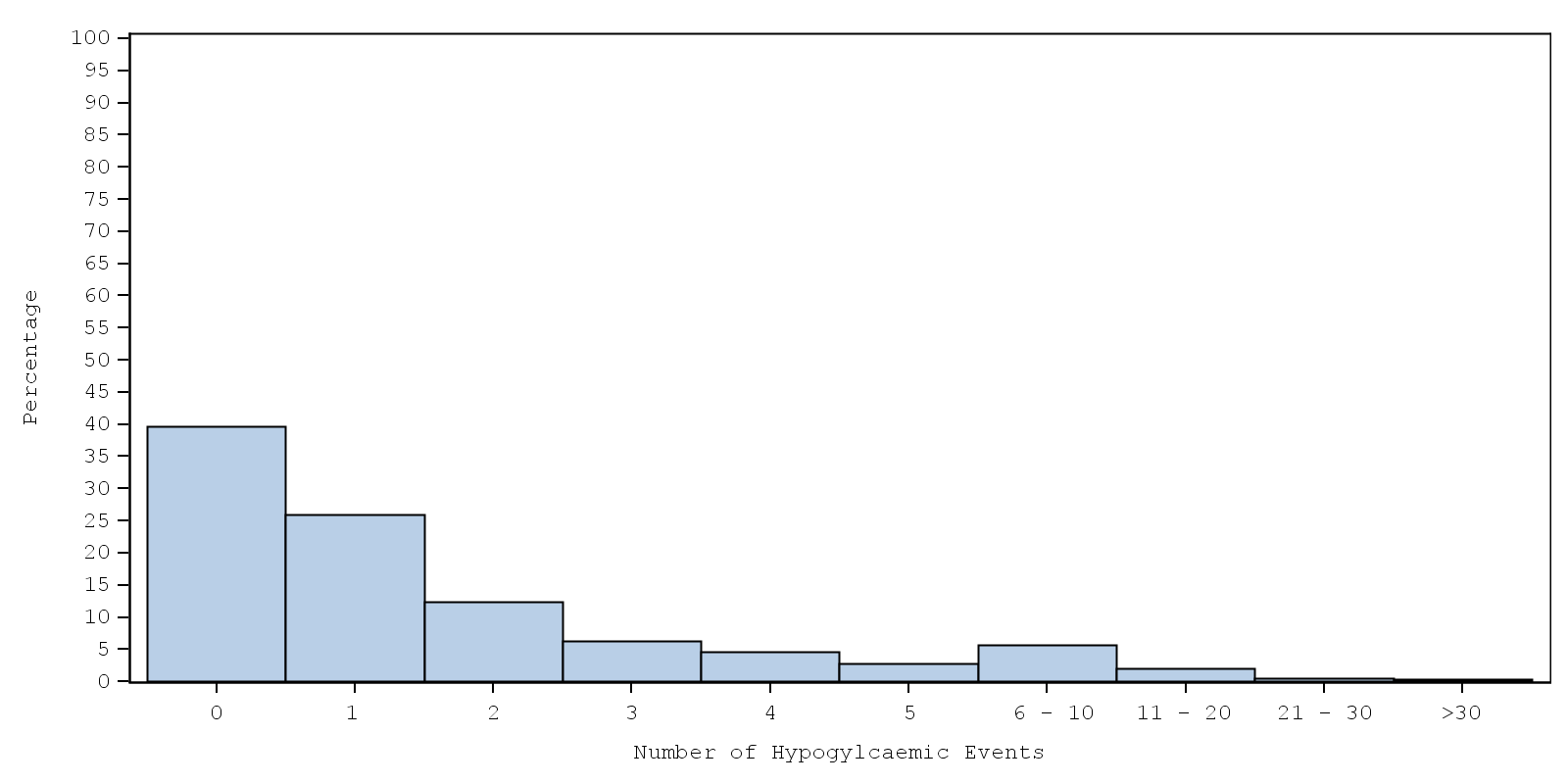


Any hypoglycaemia defined as either severe (an event requiring assistance of another person to actively administer carbohydrate, glucagon, or other resuscitative actions) or non-severe hypoglycaemia (an event managed by the patient alone), except in cases where a hypoglycaemic event was inferred from other questionnaire responses and severity was unknown.

**Distribution of Hypoglycaemic Events for Patients Experiencing Any Hypoglycaemia in the 4 Weeks After Baseline (Full Analysis Set)**


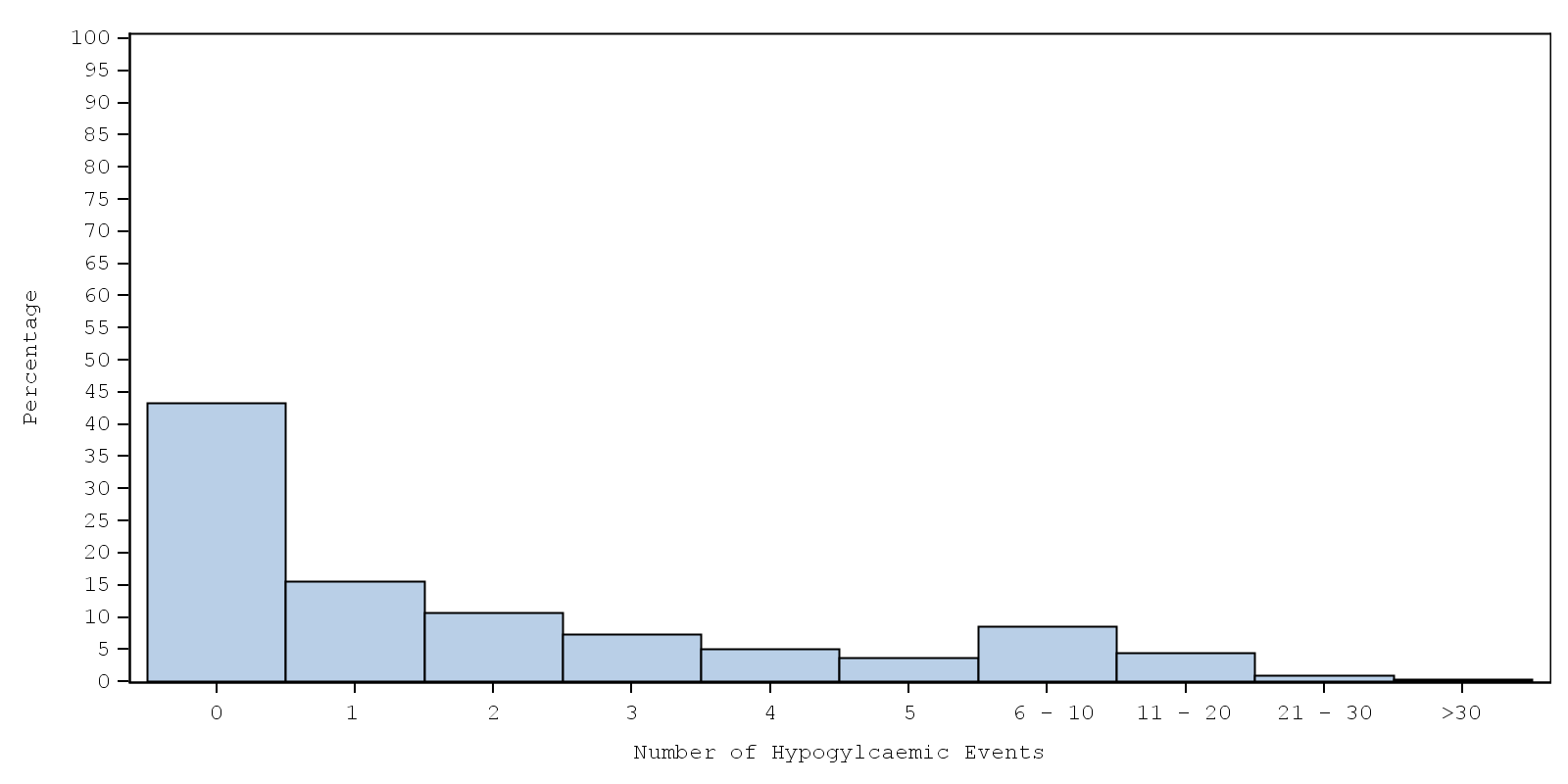


Any hypoglycaemia defined as either severe (an event requiring assistance of another person to actively administer carbohydrate, glucagon, or other resuscitative actions) or non-severe hypoglycaemia (an event managed by the patient alone), except in cases where a hypoglycaemic event was inferred from other questionnaire responses and severity was unknown.

**Distribution of Hypoglycaemic Events for Patients Experiencing Severe Hypoglycaemia in the 6 Months Before Baseline (Full Analysis Set)**


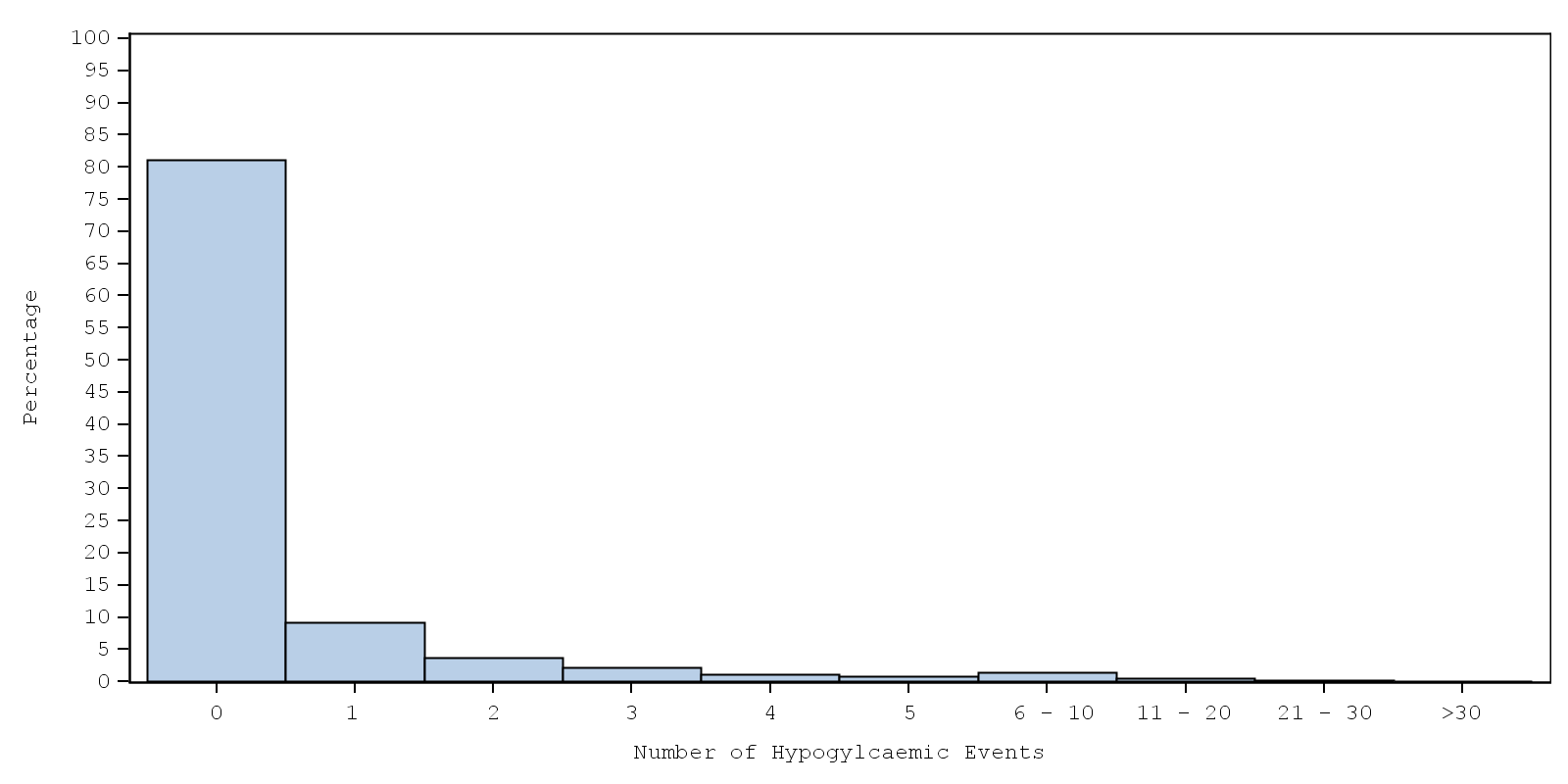


Severe hypoglycaemia defined as an event requiring assistance of another person to actively administer carbohydrate, glucagon, or other resuscitative actions.

**Distribution of Hypoglycaemic Events for Patients Experiencing Severe Hypoglycaemia in the 4 Weeks After Baseline (Full Analysis Set)**


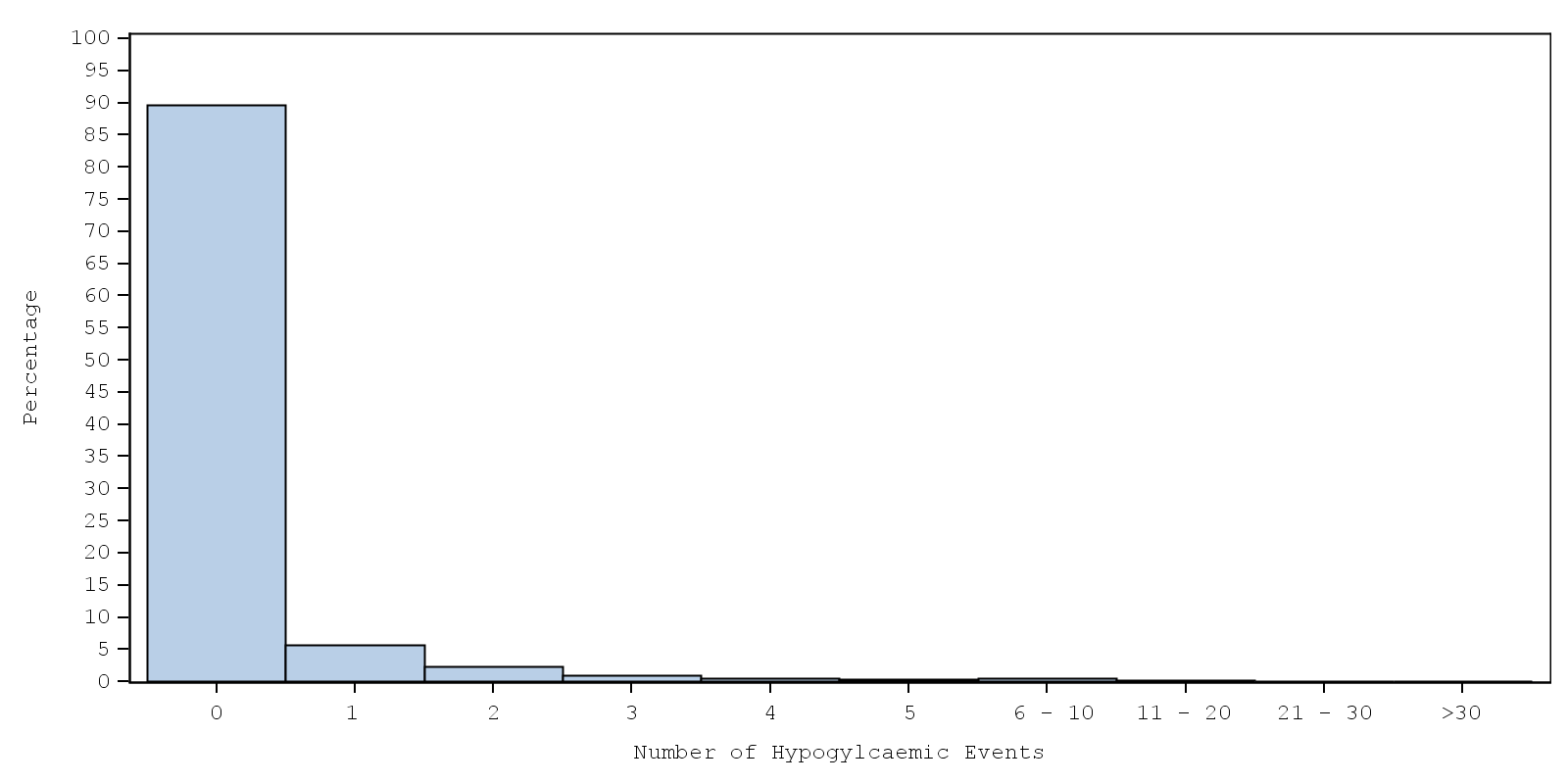


Severe hypoglycaemia defined as an event requiring assistance of another person to actively administer carbohydrate, glucagon, or other resuscitative actions.

**Distribution of Hypoglycaemic Events for Patients Experiencing Non-severe Hypoglycaemia in the 4 Weeks Before Baseline (Full Analysis Set)**


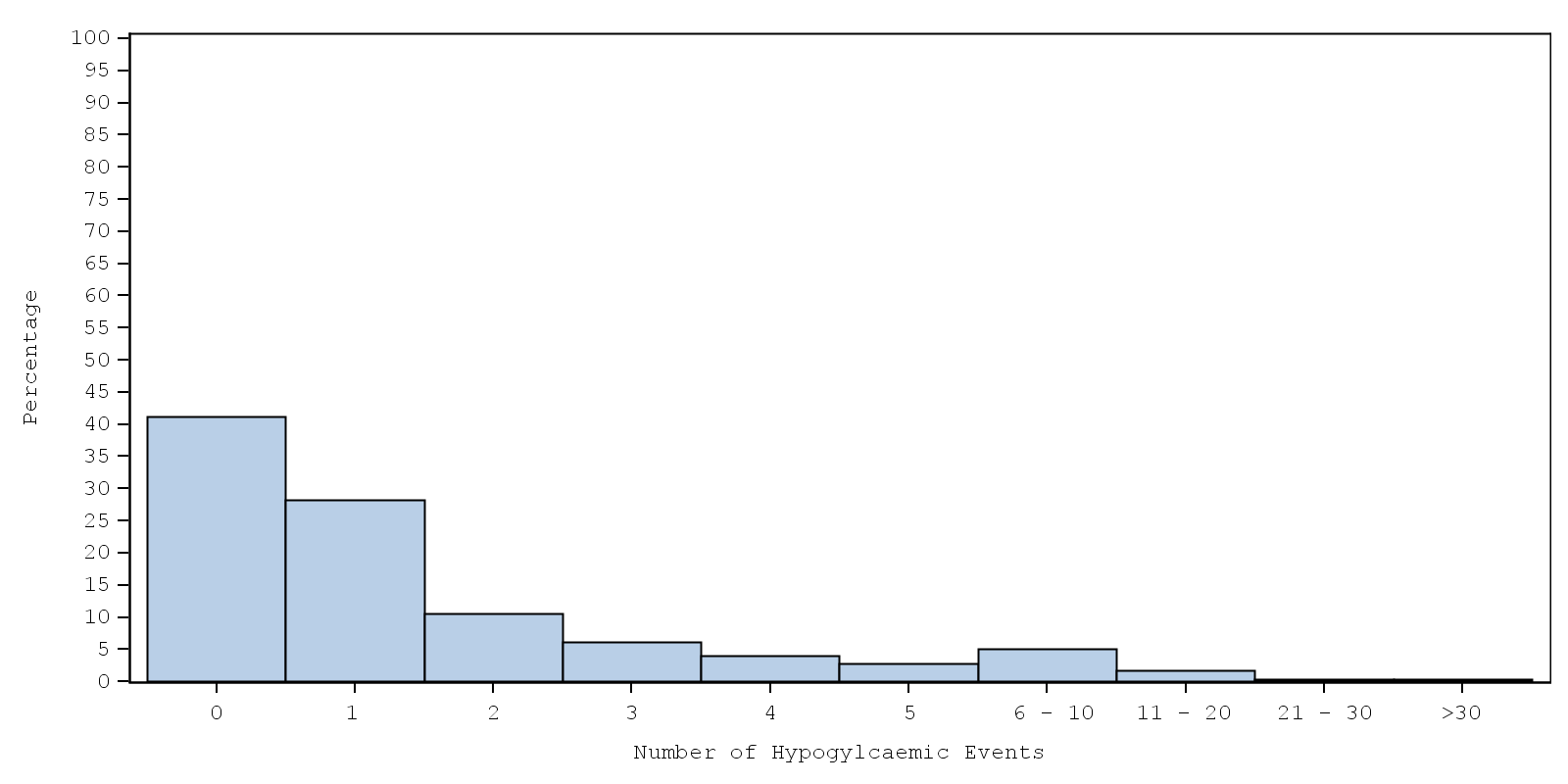


Non-severe hypoglycaemia defined as an event managed by the patient alone.

**Distribution of Hypoglycaemic Events for Patients Experiencing Non-severe Hypoglycaemia in the 4 Weeks After Baseline**


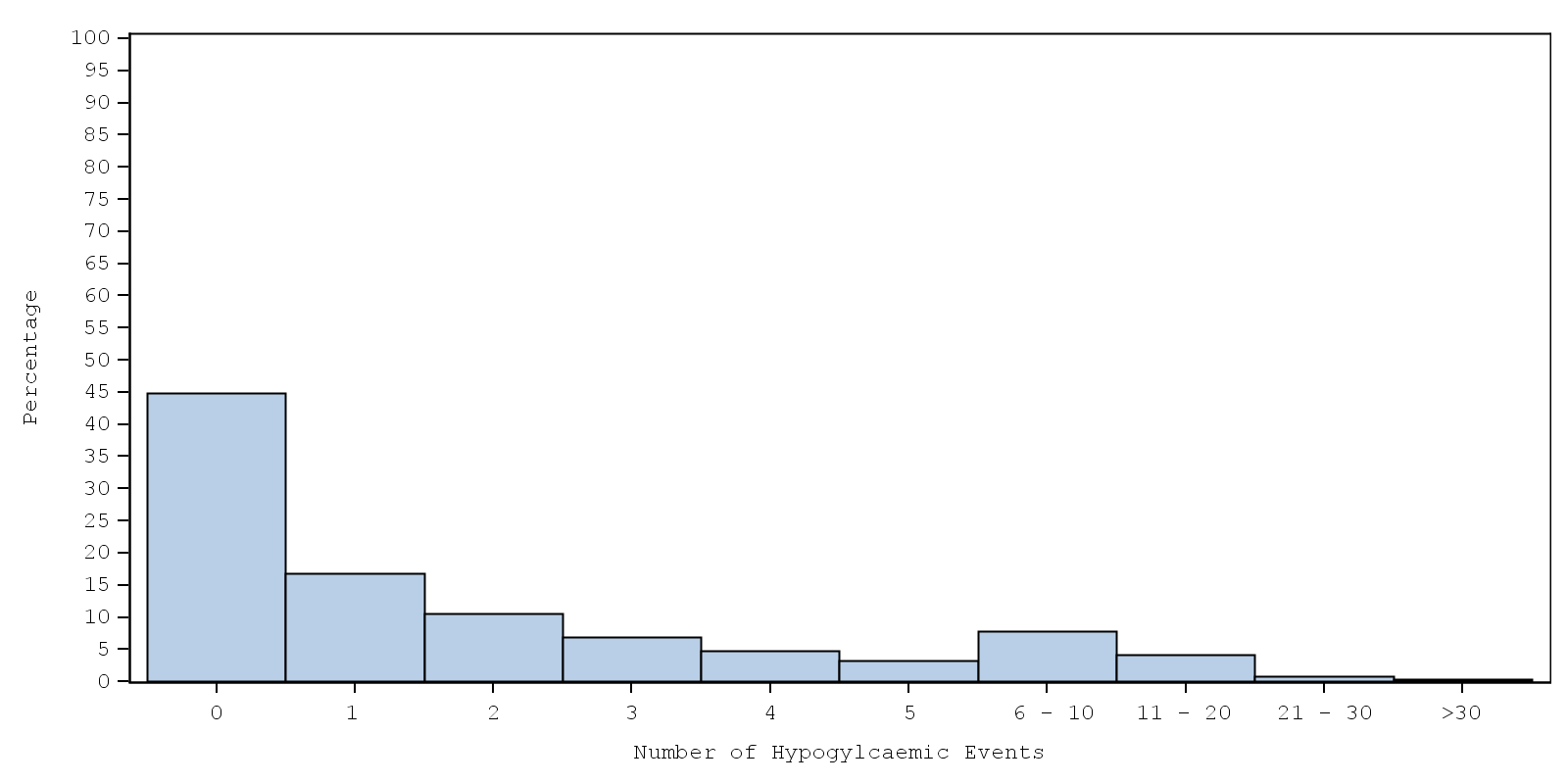


Non-severe hypoglycaemia defined as an event managed by the patient alone.

**Distribution of Hypoglycaemic Events for Patients Experiencing Nocturnal Hypoglycaemia in the 4 Weeks Before Baseline (Full Analysis Set)**


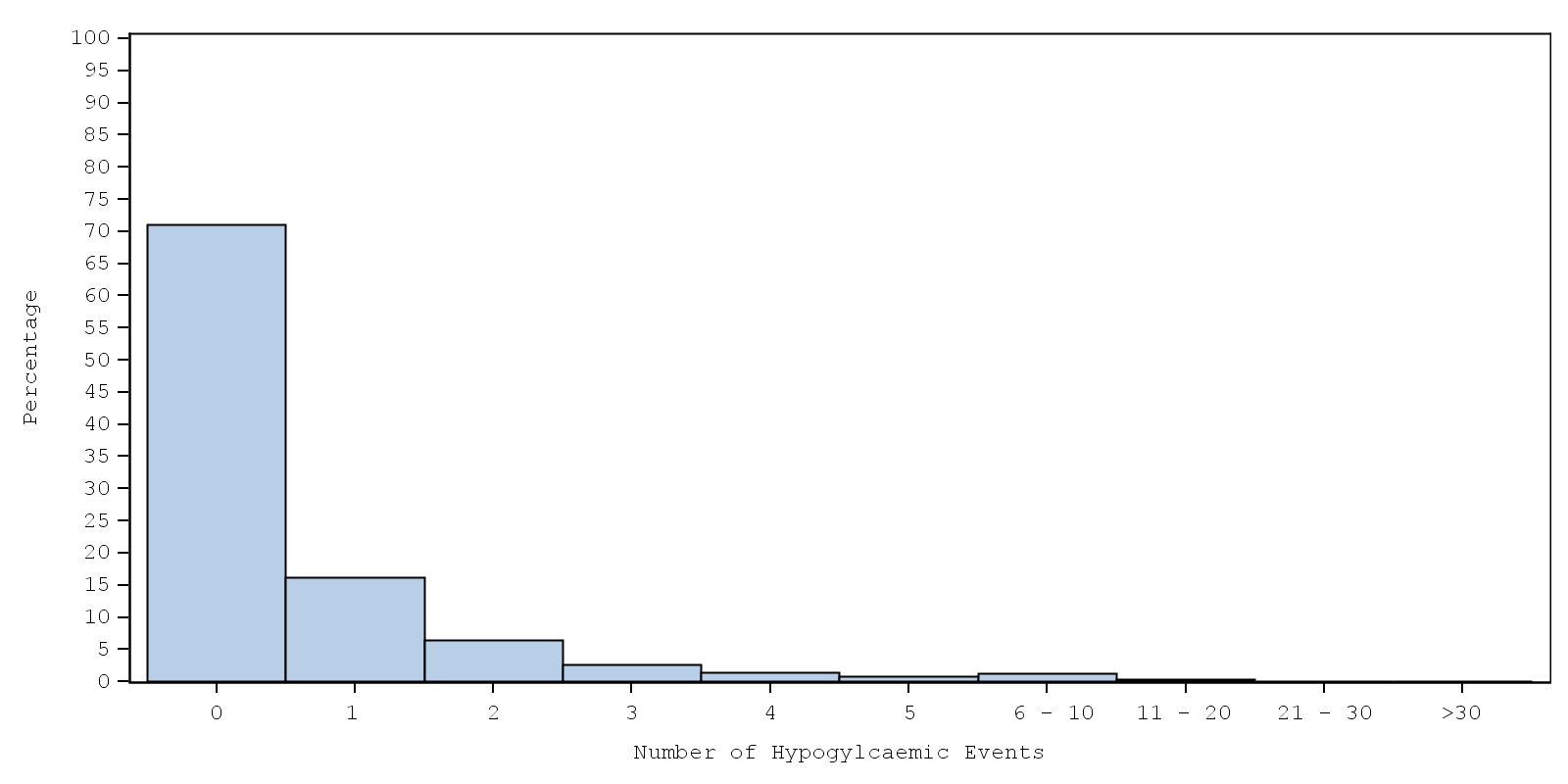


Nocturnal hypoglycaemia defined as an event occurring between the hours of midnight and 6am.

**Distribution of Hypoglycaemic Events for Patients Experiencing Nocturnal Hypoglycaemia in the 4 Weeks After Baseline (Full Analysis Set)**


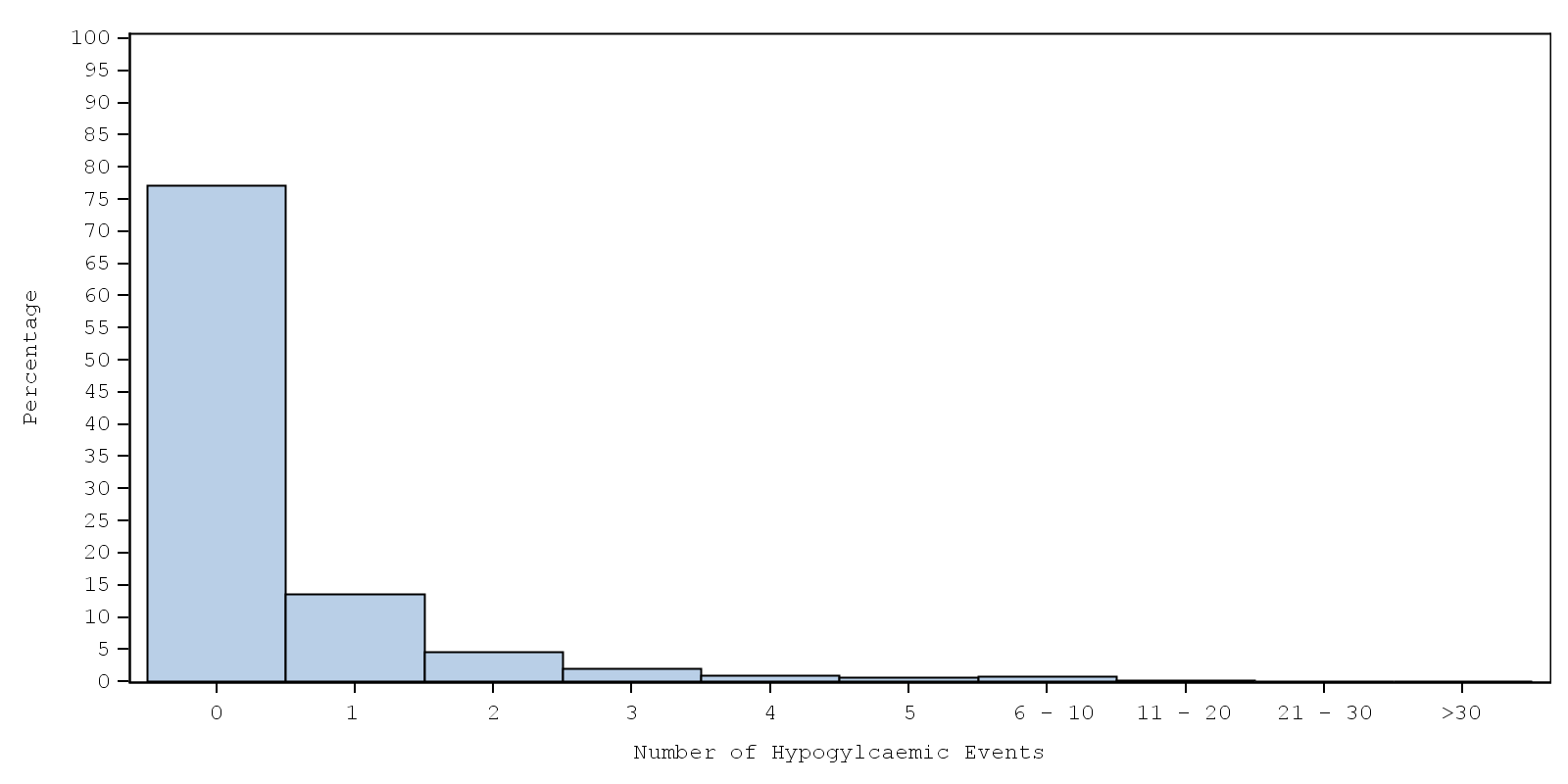


Nocturnal hypoglycaemia defined as an event occurring between the hours of midnight and 6am.
