# Supplementary material for: Rates and predictors of hypoglycaemia in 27 585 people from 24 countries with insulin‐treated type 1 and type 2 diabetes: the global HAT study
Source: Diabetes Obes Metab. 2016 Jun 20;18(9):907–15. doi: 10.1111/dom.12689 (PMC5031206; doi:10.1111/dom.12689)
Supplement: Supplementary file 2 — File S2. Baseline characteristics by region of patients with type 1 diabetes. [file DOM-18-907-s002.docx]

          Patients with more Hypoglycaemic Events recorded in the Patient Diary compared to Part 2 SAQ in the 4 weeks after,

                                                 Baseline by Diabetes Type and Overall

                                                          (Full Analysis Set)

_____________________________________________________________________________________________________________________________________

T1DM T2DM Overall

(N=7108) (N=18518) (N=25626)

_____________________________________________________________________________________________________________________________________

Number of patients recording more hypoglycaemic 2024 (28.5) 2679 (14.5) 4703 (18.4)

events using the Patient Diary than the Part 2 SAQ [n (%)]

Number of patients recording the following differences:

1 630 (8.9) 1319 (7.1) 1949 (7.6)

2 320 (4.5) 522 (2.8) 842 (3.3)

3 201 (2.8) 293 (1.6) 494 (1.9)

4 150 (2.1) 154 (0.8) 304 (1.2)

5 128 (1.8) 85 (0.5) 213 (0.8)

6 - 10 301 (4.2) 180 (1.0) 481 (1.9)

11 - 20 201 (2.8) 80 (0.4) 281 (1.1)

>20 93 (1.3) 46 (0.2) 139 (0.5)

_____________________________________________________________________________________________________________________________________

Note: Percentages based on the number of patients with evaluable data. NC = Not Calculable.

Any hypoglycaemia defined as either severe (an event requiring assistance of another person to actively administer carbohydrate

glucagon, or other resuscitative actions) or non-severe hypoglycaemia (an event managed by the patient alone), except in cases

where a hypoglycaemic event was inferred from other questionnaire responses and severity was unknown.

          Patients with less Hypoglycaemic Events recorded in the Patient Diary compared to Part 2 SAQ in the 4 weeks after,

                                                 Baseline by Diabetes Type and Overall

                                                          (Full Analysis Set)

_____________________________________________________________________________________________________________________________________

T1DM T2DM Overall

(N=7108) (N=18518) (N=25626)

_____________________________________________________________________________________________________________________________________

Number of patients recording less hypoglycaemic 789 (11.1) 1299 (7.0) 2088 (8.1)

events using the Patient Diary than the Part 2 SAQ [n (%)]

Number of patients recording the following differences:

1 442 (6.2) 898 (4.8) 1340 (5.2)

2 147 (2.1) 205 (1.1) 352 (1.4)

3 64 (0.9) 79 (0.4) 143 (0.6)

4 36 (0.5) 39 (0.2) 75 (0.3)

5 14 (0.2) 22 (0.1) 36 (0.1)

6 - 10 61 (0.9) 41 (0.2) 102 (0.4)

11 - 20 21 (0.3) 14 (<0.1) 35 (0.1)

>20 4 (<0.1) 1 (<0.1) 5 (<0.1)

_____________________________________________________________________________________________________________________________________

Note: Percentages based on the number of patients with evaluable data. NC = Not Calculable.

Any hypoglycaemia defined as either severe (an event requiring assistance of another person to actively administer carbohydrate

glucagon, or other resuscitative actions) or non-severe hypoglycaemia (an event managed by the patient alone), except in cases

where a hypoglycaemic event was inferred from other questionnaire responses and severity was unknown.

          Patients with more Severe Hypoglycaemic Events recorded in the Patient Diary compared to Part 2 SAQ in the 4 weeks

                                              After Baseline by Diabetes type and Overall

                                                          (Full Analysis Set)

_____________________________________________________________________________________________________________________________________

T1DM T2DM Overall

(N=7108) (N=18518) (N=25626)

_____________________________________________________________________________________________________________________________________

Number of patients recording more severe hypoglycaemic 449 (6.3) 789 (4.3) 1238 (4.8)

events using the Patient Diary than the Part 2 SAQ [n (%)]

Number of patients recording the following differences:

1 262 (3.7) 485 (2.6) 747 (2.9)

2 94 (1.3) 169 (0.9) 263 (1.0)

3 33 (0.5) 47 (0.3) 80 (0.3)

4 17 (0.2) 22 (0.1) 39 (0.2)

5 10 (0.1) 18 (<0.1) 28 (0.1)

6 - 10 23 (0.3) 31 (0.2) 54 (0.2)

11 - 20 8 (0.1) 9 (<0.1) 17 (<0.1)

>20 2 (<0.1) 8 (<0.1) 10 (<0.1)

_____________________________________________________________________________________________________________________________________

Note: Percentages based on the number of patients with evaluable data. NC = Not Calculable.

Severe hypoglycaemia defined as an event requiring assistance of another person to actively administer carbohydrate, glucagon

or other resuscitative actions.

          Patients with less Severe Hypoglycaemic Events recorded in the Patient Diary compared to Part 2 SAQ in the 4 weeks

                                              After Baseline by Diabetes type and Overall

                                                          (Full Analysis Set)

_____________________________________________________________________________________________________________________________________

T1DM T2DM Overall

(N=7108) (N=18518) (N=25626)

_____________________________________________________________________________________________________________________________________

Number of patients recording less severe hypoglycaemic 275 (3.9) 389 (2.1) 664 (2.6)

events using the Patient Diary than the Part 2 SAQ [n (%)]

Number of patients recording the following differences:

1 169 (2.4) 250 (1.4) 419 (1.6)

2 56 (0.8) 76 (0.4) 132 (0.5)

3 14 (0.2) 34 (0.2) 48 (0.2)

4 17 (0.2) 14 (<0.1) 31 (0.1)

5 1 (<0.1) 5 (<0.1) 6 (<0.1)

6 - 10 8 (0.1) 6 (<0.1) 14 (<0.1)

11 - 20 9 (0.1) 4 (<0.1) 13 (<0.1)

>20 1 (<0.1) 0 1 (<0.1)

_____________________________________________________________________________________________________________________________________

Note: Percentages based on the number of patients with evaluable data. NC = Not Calculable.

Severe hypoglycaemia defined as an event requiring assistance of another person to actively administer carbohydrate, glucagon

or other resuscitative actions.

        Patients with more Non-Severe Hypoglycaemic events recorded in the Patient Diary compared to Part 2 SAQ in the 4 weeks

                                              After Baseline by Diabetes Type and Overall

                                                          (Full Analysis Set)

_____________________________________________________________________________________________________________________________________

T1DM T2DM Overall

(N=7108) (N=18518) (N=25626)

_____________________________________________________________________________________________________________________________________

Number of patients recording more non-severe hypoglycaemic 1990 (28.0) 2509 (13.5) 4499 (17.6)

events using the Patient Diary than the Part 2 SAQ [n (%)]

Number of patients recording the following differences:

1 611 (8.6) 1225 (6.6) 1836 (7.2)

2 321 (4.5) 513 (2.8) 834 (3.3)

3 201 (2.8) 295 (1.6) 496 (1.9)

4 147 (2.1) 138 (0.7) 285 (1.1)

5 128 (1.8) 85 (0.5) 213 (0.8)

6 - 10 293 (4.1) 153 (0.8) 446 (1.7)

11 - 20 201 (2.8) 64 (0.3) 265 (1.0)

>20 88 (1.2) 36 (0.2) 124 (0.5)

_____________________________________________________________________________________________________________________________________

Note: Percentages based on the number of patients with evaluable data. NC = Not Calculable.

Non-severe hypoglycaemia defined as an event managed by the patient alone.

        Patients with less Non-Severe Hypoglycaemic events recorded in the Patient Diary compared to Part 2 SAQ in the 4 weeks

                                              After Baseline by Diabetes Type and Overall

                                                          (Full Analysis Set)

_____________________________________________________________________________________________________________________________________

T1DM T2DM Overall

(N=7108) (N=18518) (N=25626)

_____________________________________________________________________________________________________________________________________

Number of patients recording less non-severe hypoglycaemic 863 (12.1) 1471 (7.9) 2334 (9.1)

events using the Patient Diary than the Part 2 SAQ [n (%)]

Number of patients recording the following differences:

1 491 (6.9) 1056 (5.7) 1547 (6.0)

2 163 (2.3) 209 (1.1) 372 (1.5)

3 66 (0.9) 83 (0.4) 149 (0.6)

4 42 (0.6) 43 (0.2) 85 (0.3)

5 19 (0.3) 28 (0.2) 47 (0.2)

6 - 10 59 (0.8) 42 (0.2) 101 (0.4)

11 - 20 20 (0.3) 10 (<0.1) 30 (0.1)

>20 3 (<0.1) 0 3 (<0.1)

_____________________________________________________________________________________________________________________________________

Note: Percentages based on the number of patients with evaluable data. NC = Not Calculable.

Non-severe hypoglycaemia defined as an event managed by the patient alone.
