# Supplementary material for: Rates and predictors of hypoglycaemia in 27 585 people from 24 countries with insulin‐treated type 1 and type 2 diabetes: the global HAT study
Source: Diabetes Obes Metab. 2016 Jun 20;18(9):907–15. doi: 10.1111/dom.12689 (PMC5031206; doi:10.1111/dom.12689)
Supplement: Supplementary file 3 — Table S1. Patient disposition by country. Table S2. Baseline characteristics by region of patients with type 1 diabetes. Table S3. Baseline characteristics by region of patients with type 2 diabetes. Table S4. Severe hypoglycaemia rates during the prospective period by geographic region. [file DOM-18-907-s003.doc]

# Supplementary information

Supplemental Table S1*–*Patient disposition by country

|  | **Diabetes type** | | **Sections completed, n (%)** | | |
| --- | --- | --- | --- | --- | --- |
|  | T1DM | T2DM | SAQ Part 1 | SAQ Part 2 | PD |
| **Argentina** | 433 | 823 | 1256 | 992 (79.0) | 994 (79.1) |
| **Austria** | 222 | 284 | 506 | 409 (80.8) | 393 (77.7) |
| **Bulgaria** | 226 | 311 | 537 | 536 (99.8) | 536 (99.8) |
| **Canada** | 183 | 315 | 498 | 403 (80.9) | 410 (82.3) |
| **Croatia** | 104 | 211 | 315 | 299 (94.9) | 294 (93.3) |
| **Czech Republic** | 641 | 831 | 1472 | 1427 (96.9) | 787 (53.5) |
| **Denmark** | 458 | 351 | 809 | 432 (53.4) | 432 (53.4) |
| **Finland** | 301 | 105 | 406 | 324 (79.8) | 326 (80.3) |
| **Germany** | 811 | 1619 | 2430 | 2056 (84.6) | 2044 (84.1) |
| **Hungary** | 449 | 1218 | 1667 | 1590 (95.4) | 1587 (95.2) |
| **India** | 112 | 2808 | 2920 | 2884 (98.8) | 2873 (98.4) |
| **Israel** | 545 | 980 | 1525 | 1351 (88.6) | 1350 (88.5) |
| **Lebanon** | 250 | 655 | 905 | 851 (94.0) | 837 (92.5) |
| **Malaysia** | 114 | 1039 | 1153 | 1143 (99.1) | 1142 (99.0) |
| **Mexico** | 98 | 837 | 935 | 899 (96.1) | 899 (96.1) |
| **The Netherlands** | 167 | 531 | 698 | 633 (90.7) | 627 (89.8) |
| **Poland** | 695 | 1753 | 2448 | 2387 (97.5) | 1462 (59.7) |
| **Romania** | 365 | 825 | 1190 | 1131 (95.0) | 1131 (95.0) |
| **Russia** | 618 | 737 | 1355 | 1336 (98.6) | 1326 (97.9) |
| **Saudi Arabia** | 329 | 1438 | 1767 | 1720 (97.3) | 1484 (84.0) |
| **Serbia and Montenegro** | 394 | 670 | 1064 | 1060 (99.6) | 1059 (99.5) |
| **Slovakia** | 177 | 323 | 500 | 492 (98.4) | 484 (96.8) |
| **Slovenia** | 84 | 227 | 311 | 307 (98.7) | 307 (98.7) |
| **Sweden** | 246 | 672 | 918 | 843 (91.8) | 843 (91.8) |
| **Total** | 8022 | 19563 | 27585 | 25505 (92.5) | 23627 (85.7) |

SAQ, self-assessment questionnaire; PD, patient diary; T1DM, type 1 diabetes; T2DM, type 2 diabetes

Supplemental Table S2*–*Baseline characteristics by region of patients with type 1 diabetes

| **Characteristic** | **Northern Europe/**  **Canada**  **(*n* = 2388)** | **Eastern Europe**  **(*n* = 3135)** | **Latin America**  **(*n* = 531)** | **Middle East**  **(*n* = 1124)** | **Russia**  **(*n* = 618)** | **SE Asia**  **(*n* = 226)** |
| --- | --- | --- | --- | --- | --- | --- |
| **Mean age, years (SD)** | 46.3 (15.3) | 42.0 (14.0) | 40.1 (16.1) | 38.0 (15.7) | 37.5 (13.1) | 36.8 (14.3) |
| Median | 46.0 | 40.0 | 37.0 | 34.0 | 36.0 | 33.0 |
| Upper quartile, lower quartile | 58.0, 34.0 | 52.0, 31.0 | 50.0, 27.0 | 49.0, 25.0 | 46.0, 27.0 | 45.0, 25.0 |
| **Sex male/female, %** | 49.6/50.4 | 48.3/51.7 | 39.8/60.2 | 50.6/49.4 | 42.4/57.6 | 48.2/51.8 |
| **Duration of diabetes, years (SD)** | 21.3 (13.7) | 16.8 (11.2) | 16.6 (11.7) | 15.3 (10.5) | 14.5 (9.5) | 11.6 (8.0) |
| Median | 19.0 | 15.0 | 15.0 | 13.0 | 13.0 | 10.0 |
| Upper quartile, lower quartile | 30.0, 10.0 | 24.0, 8.0 | 22.0, 7.0 | 20.0, 8.0 | 20.0, 7.0 | 15.0, 5.0 |
| **Duration of insulin use, years (SD)** | 20.7 (13.8) | 16.3 (11.2) | 15.7 (11.8) | 14.2 (10.3) | 14.3 (9.5) | 9.9 (8.3) |
| Median | 18.0 | 14.0 | 14.0 | 12.0 | 13.0 | 8.0 |
| Upper quartile, lower quartile | 30.0, 10.0 | 23.0, 7.0 | 21.0, 7.0 | 20.0, 7.0 | 20.0, 7.0 | 15.0, 3.0 |
| **HbA_1c_, mmol/mol (SD)** | 62.3 (14.5) | 61.0 (15.7) | 65.6 (19.2) | 65.6 (16.4) | 64.6 (18.5) | 73.3 (21.0) |
| **HbA_1c_, %*** | 7.9 | 7.7 | 8.2 | 8.2 | 8.1 | 8.9 |

Data are presented as mean (SD) unless otherwise stated. Analyses based on full analysis set. *n* is defined as total number of patients participating. *Calculated, not measured. SD, standard deviation.

Supplementary Table S3*–*Baseline characteristics by region of patients with type 2 diabetes

| **Characteristic** | **Northern Europe/**  **Canada**  **(*n* = 3877)** | **Eastern Europe**  **(*n* = 6369)** | **Latin America**  **(*n* = 1660)** | **Middle East**  **(*n* = 3073)** | **Russia**  **(*n* = 737)** | **SE Asia**  **(*n* = 3847)** |
| --- | --- | --- | --- | --- | --- | --- |
| **Mean age, years (SD)** | 65.6 (10.5) | 62.5 (9.3) | 61.2 (11.9) | 58.0 (11.5) | 60.3 (8.9) | 55.3 (10.4) |
| Median | 67.0 | 63.0 | 62.0 | 58.0 | 60.0 | 56.0 |
| Upper quartile, lower quartile | 73.0, 59.0 | 69.0, 57.0 | 70.0, 54.0 | 66.0, 50.0 | 66.0, 55.0 | 62.0, 49.0 |
| **Sex male/female, %** | 56.3/43.7 | 48.7/51.3 | 43.6/56.4 | 56.4/43.6 | 28.4/71.6 | 60.5/39.5 |
| **Duration of diabetes, years (SD)** | 15.4 (8.9) | 13.6 (7.9) | 14.6 (9.0) | 14.9 (8.3) | 12.5 (7.3) | 10.9 (6.9) |
| Median | 14.0 | 12.0 | 13.0 | 14.0 | 11.0 | 10.0 |
| Upper quartile, lower quartile | 20.0, 10.0 | 18.0, 8.0 | 20.0, 8.0 | 20.0, 9.0 | 17.0, 7.0 | 15.0, 6.0 |
| **Duration of insulin use, years (SD)** | 8.3 (6.7) | 7.1 (5.8) | 5.7 (5.3) | 5.6 (5.3) | 6.1 (4.7) | 4.0 (3.5) |
| Median | 7.0 | 5.0 | 4.0 | 4.0 | 5.0 | 3.0 |
| Upper quartile, lower quartile | 12.0, 3.0 | 10.0, 3.0 | 7.0, 2.0 | 7.0, 2.0 | 8.0, 3.0 | 5.0, 2.0 |
| **HbA_1c_, mmol/mol (SD)** | 60.3 (14.5) | 60.9 (15.0) | 64.9 (18.8) | 68.6 (16.0) | 64.3 (15.2) | 71.8 (17.0) |
| **HbA_1c_, %*** | 7.7 | 7.7 | 8.1 | 8.4 | 8.0 | 8.7 |

Data are presented as mean (SD) unless otherwise stated. Analyses based on full analysis set. *n* is defined as total number of patients participating. *Calculated, not measured. SD, standard deviation.

Supplementary Table S4*–*Severe hypoglycemia rates during the prospective period by geographic region

|  | **Type 1 diabetes** | | **Type 2 diabetes** | |
| --- | --- | --- | --- | --- |
| **Geographic region** | Severe hypoglycemia rate  (events PPY) | 95% CI | Severe hypoglycemia rate  (events PPY) | 95% CI |
| **Global** | 4.9 | 4.7–5.1 | 2.5 | 2.4–2.5 |
| **Northern Europe/Canada** | 3.4 | 3.1–3.7 | 1.3 | 1.2–1.5 |
| **Eastern Europe** | 4.5 | 4.3–4.8 | 2.2 | 2.1–2.4 |
| **Latin America** | 10.8 | 9.7–12.0 | 3.7 | 3.3–4.1 |
| **Middle East** | 6.7 | 6.1–7.3 | 2.4 | 2.2–2.6 |
| **Russia** | 5.3 | 4.7–6.0 | 2.3 | 2.0–2.8 |
| **SE Asia** | 2.1 | 1.4–2.8 | 3.4 | 3.2–3.7 |

Data are presented as mean (95% CI) unless otherwise stated. Analyses based on completers analysis set. PPY, per patient-year.

Supplementary Table S5–Hypoglycemia rates during the prospective period by age group

|  | **Hypoglycemic events PPY (95%CI)** | |
| --- | --- | --- |
|  | Type 1 diabetes | Type 2 diabetes |
| **Any hypoglycemia** |  |  |
| All ages | 73.306 (72.588, 74.030) | 19.349 (19.121, 19.580) |
| 18-30 | 78.946 (77.504, 80.408) | 24.524 (21.488, 27.868) |
| 31-40 | 76.543 (75.017, 78.093) | 21.182 (19.920, 22.504) |
| 41-53 | 72.662 (71.235, 74.109) | 19.183 (18.674, 19.703) |
| >53 | 65.122 (63.784, 66.481) | 19.262 (19.001, 19.526) |
| **Severe hypoglycemia** |  |  |
| All ages | 4.903 (4.719, 5.093) | 2.458 (2.377, 2.541) |
| 18-30 | 5.114 (4.752, 5.496) | 2.713 (1.772, 3.976) |
| 31-40 | 4.976 (4.592, 5.383) | 3.675 (3.161, 4.250) |
| 41-53 | 5.180 (4.804, 5.577) | 2.725 (2.535, 2.926) |
| >53 | 4.346 (4.005, 4.708) | 2.329 (2.239, 2.423) |
| **Non-severe hypoglycemia** |  |  |
| All ages | 68.617 (67.922, 69.318) | 17.017 (16.802, 17.235) |
| 18-30 | 73.871 (72.476, 75.286) | 21.811 (18.954, 24.977) |
| 31-40 | 71.705 (70.227, 73.207) | 17.534 (16.387, 18.742) |
| 41-53 | 67.673 (66.295, 69.072) | 16.496 (16.024, 16.979) |
| >53 | 61.217 (59.915, 62.540) | 17.087 (16.840, 17.337) |
| **Nocturnal hypoglycemia** |  |  |
| All ages | 11.273 (10.987, 11.564) | 3.668 (3.566, 3.771) |
| 18-30 | 12.175 (11.604, 12.766) | 3.881 (2.718, 5.373) |
| 31-40 | 11.804 (11.199, 12.433) | 3.569 (3.054, 4.146) |
| 41-53 | 10.882 (10.324, 11.462) | 3.512 (3.292, 3.742) |
| >53 | 10.210 (9.671, 10.772) | 3.712 (3.594, 3.832) |

Any hypoglycemia defined as either severe (an event requiring assistance of another person to actively administer carbohydrate glucagon, or other resuscitative actions) or non-severe hypoglycemia (an event managed by the patient alone), except in cases where a hypoglycemic event was inferred from other questionnaire responses and severity was unknown. Severe hypoglycemia defined as an event requiring assistance of another person to actively administer carbohydrate, glucagon or other resuscitative actions. Non-severe hypoglycemia defined as an event managed by the patient alone. Nocturnal hypoglycemia defined as an event occurring between the hours of midnight and 6am. If a patient recorded more hypoglycemic events using the Patient Diary than the Part 2 SAQ, their Patient Diary was used to calculate incidence 4 weeks after baseline. CI, confidence interval; PPY, per patient-year.
